# Designing Nonflammable Liquid Electrolytes for Safe Li‐Ion Batteries

**DOI:** 10.1002/adma.202312451

**Published:** 2024-05-07

**Authors:** Jing Xie, Yi‐Chun Lu

**Affiliations:** ^1^ Electrochemical Energy and Interfaces Laboratory Department of Mechanical and Automation Engineering The Chinese University of Hong Kong Hong Kong SAR 999077 China

**Keywords:** liquid electrolyte, lithium‐ion batteries, nonflammable electrolyte

## Abstract

Li‐ion batteries are essential technologies for electronic products in the daily life. However, serious fire safety concerns that are closely associated with the flammable liquid electrolyte remains a key challenge. Tremendous effort has been devoted to designing nonflammable liquid electrolytes. It is critical to gain comprehensive insights into nonflammability design and inspire more efficient approaches for building safer Li‐ion batteries. This review presents current mechanistic understanding of safety issues and discusses state‐of‐the‐art nonflammable liquid electrolytes design for Li‐ion batteries based on molecule, solvation, and battery compatibility level. Various safety test methods are discussed for reliable safety risk evaluation. Finally, the challenges and perspectives of the nonflammability design for Li‐ion electrolytes are summarized.

## Graphical abstract

Current mechanistic understanding of safety issues and discussions on state‐of‐the‐art nonflammable liquid electrolytes design for Li‐ion batteries is summarized based on the level of molecule, solvation, and battery compatibility. Typical methods for safety tests are presented to evaluate fire risk of battery. Finally, the challenges and perspectives of developing nonflammability for Li‐ion electrolyte are discussed.

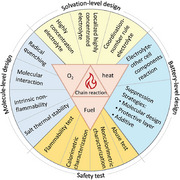

## 1 Introduction

Li‐ion battery is an indispensable technology in our daily life considering its high energy density (250–400 Wh kg^−1^), long cycle life, good rate capability, and cost compared with other battery technology.^[^ ^1^ ^]^ The demand for Li‐ion battery grows rapidly in portable electronics, electric vehicles, and grid scale energy storage.^[^ ^2^ ^]^ Commercial Li‐ion battery typically consists of graphite or graphite‐Si/SiO*_x_* composite anode,^[^ ^3^ ^]^ oxide cathode (layered, e.g., LiCoO_2_, LiNi_1−_ *_y_*_−_ *_z_* Mn*_y_* Co*_z_* O_2_ (NMC); spinel, e.g., LiMn_2_O_4_; polyanion families, e.g., LiFePO_4_),^[^ ^4^ ^]^ and polyethylene (PE)/polypropylene (PP) separator immersed with nonaqueous electrolyte.^[^ ^5^ ^]^ Traditional nonaqueous electrolyte is composed of LiPF_6_ salt, a mixture solvent of ethylene carbonate (EC) and linear carbonates (e.g., dimethyl carbonate (DMC), ethyl methyl carbonate (EMC), diethyl carbonate (DEC)).^[^ ^6^ ^]^ Usually a self‐passivating solid‐electrolyte interphase (SEI) evolves on the anode surface during cycling to suppress continuous electrolyte decomposition and support a long cycle life.^[^ ^7^ ^]^

However, fire hazard of commercial Li‐ion battery has raised serious safety concerns, which is considered to be highly correlated with the usage of flammable liquid electrolyte.^[^ ^6^, ^8^ ^]^ Although solid polymer and ceramic electrolyte have been proposed as a candidate for safe battery due to their low flammability,^[^ ^9^ ^]^ liquid electrolyte is still in high demand owing to its high ionic conductivity, low interfacial resistance, and more flexibility compared with that of solid electrolyte.^[^ ^10^ ^]^ Furthermore, liquid electrolyte requires a lower manufacturing cost compared to solid electrolyte.^[^ ^11^ ^]^ Therefore, the development of nonflammable liquid electrolyte is attracting extensive attention.^[^ ^5^, ^12^ ^]^ However, the usage of currently‐reported nonflammable liquid electrolytes usually require high‐cost and toxic materials,^[^ ^13^ ^]^ compromise battery performance,^[^ ^12b^ ^]^ or fail to avoid fire at battery level.^[^ ^14^ ^]^ It remains a key challenge to achieve both battery safety and cycle stability using cost‐effective and green technologies.

This review starts with the discussion of state‐of‐the‐art mechanistic understanding of safety hazard occurs in Li‐ion batteries. Design strategies based on the level of molecule, solvation structure, and battery (compatibility among cell components) are reviewed. Typical safety tests including electrolyte flammability test, calorimetric test, noncalorimetric characterization, and abuse test are summarized to provide a more comprehensive evaluation of battery safety. Finally, conclusion and perspective are presented for designing effective nonflammable liquid electrolyte and promoting the development of safe, stable, and sustainable Li‐ion batteries.

## 2 Mechanism

**Figure 1:**
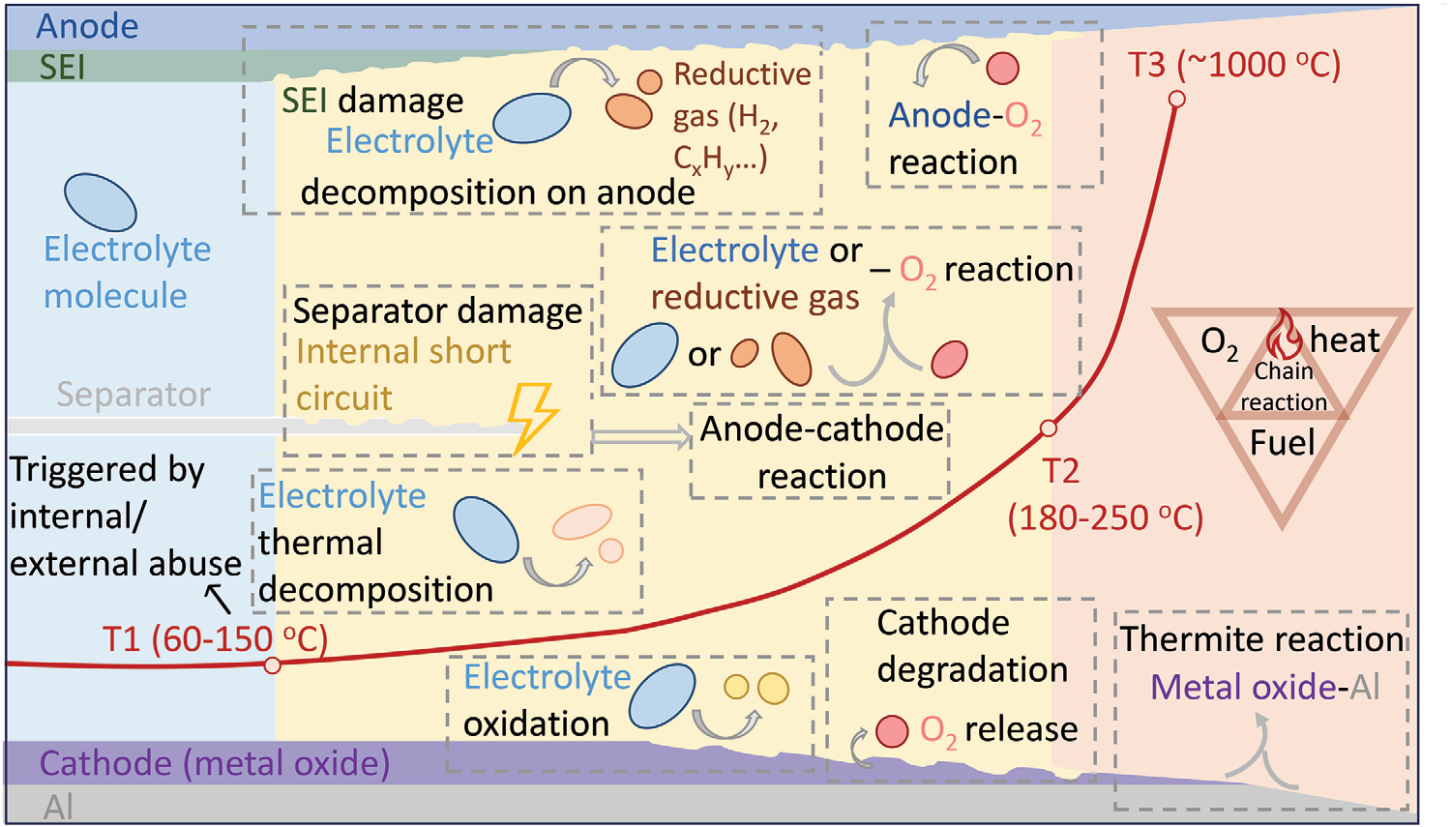
Schematic illustration of thermal runaway mechanism in Li‐ion batteries.

Thermal hazard of Li‐ion battery is usually induced by internal defects (lithium dendrite, separator flaws, etc.) or external abuse (e.g., cell crush, overheating).^[^ ^15^ ^]^ Several exothermic reactions occur subsequently inside the battery (as shown in **Figure** **1**), leading to accelerated propagations of the reaction if the generated heat exceeds the dissipated heat.^[^ ^16^ ^]^ This whole process is usually characterized by three characteristic temperatures which are defined in an adiabatic thermal runaway measurement (accelerated rate calorimetry, ARC).

T1 (60–150 °C)^[^ ^17^ ^]^ represents the onset temperature of self‐heating with a temperature rising rate of around 0.02–0.05 °C min^−1^.^[^ ^18^ ^]^ This process could last from several minutes to days.^[^ ^19^ ^]^ The main side reactions are

1. SEI breakdown owing to high temperature (>61 °C^[^ ^20^ ^]^) or physical penetration.^[^ ^16^ ^]^ Generally, SEI in commercial Li‐ion battery consists of inorganic (LiF, Li_2_CO_3_, etc.) and metastable organic components (e.g., lithium‐alkyl carbonates). The organic components are prone to decompose thermally, for example^[^ ^21^ ^]^
(1) $${\left(CH_{2}\mathrm{OC}O_{2}\mathrm{Li}\right)}_{2}\to Li_{2}CO_{3}+C_{2}H_{4}+CO_{2}+\frac{1}{2}O_{2}$$
2. Chemical reaction between electrolyte and anode (metallic or intercalated Li), resulting in generation of reductive gas (>80 °C^[^ ^22^ ^]^), for instance^[^ ^23^ ^]^
(2) $$2\mathrm{Li}+C_{3}H_{4}O_{3}\left(\mathrm{EC}\right)\to Li_{2}CO_{3}+C_{2}H_{4}$$
3. Thermal decomposition of electrolyte components (>70 °C),^[^ ^24^ ^]^ including the decomposition of LiPF_5_, which produces strong Lewis acid PF_5_ and induce decompositions of other solvent components
(3) $$\mathrm{LiP}F_{6}\to \mathrm{LiF}+PF_{5}$$
(4) $$O\;=\;C{\left(\mathrm{OR}\right)}_{2}\;\overset{PF_{5}}{\to }\;R-F,\;R^{1}-O-R^{2},\;\mathrm{alkenes},\;CO_{2}$$
4. Solvent evaporation (>80 °C^[^ ^25^ ^]^), leading to increased internal pressure within the batteries.

T2 (180–250 °C.^17^ ^]^) indicates the starting point of uncontrollable thermal runaway, it can be defined when the temperature rising rate is higher than 60 °C min^−1^.^[^ ^18^, ^26^ ^]^ Several reactions occur at this stage

1. The shrinkage of separator results in the direct contact between cathode and anode materials, leading to severe exothermic reaction.^[^ ^17^ ^]^
2. Reaction between cathode and reductive gas (e.g., C*_x_* H*_y_*, H_2_).^[^ ^22^ ^]^
3. Oxygen is released from oxide cathode due to its structure collapse, the released oxygen reacts with electrolyte,^[^ ^26^ ^]^ reductive gas, and anode.^[^ ^26^, ^27^ ^]^

T3 is the maximum temperature with a rapid temperature rising rate (for example, 10^4^ °C min^−1^ for NCM111‐based battery^[^ ^18b^ ^]^), it is related to the total energy released by the system during thermal runaway process^[^ ^17^ ^]^ and can be higher than 1000 °C in high‐energy battery.^[^ ^28^ ^]^ Main reactions are

1. Pressure inside the battery increases rapidly, leading to explosion and leakage of flammable gas, resulting in catastrophic fire with external air.^[^ ^29^ ^]^
2. Thermite reaction between Al current collector and cathode (metal oxide) occurs and generates large amount of heat.^[^ ^30^ ^]^

Notably, the temperature of each reaction or stage varies depending on the battery condition including states of charges, battery types, and sizes.^[^ ^31^ ^]^ In addition, the above listed exothermic reactions may occur simultaneously, or vary in order depending on environmental condition.^[^ ^31^ ^]^ For example, thermal runaway is usually induced by short circuit, which is caused by the collapse of separator. However, it is suggested that thermal runaway of high‐energy batteries is triggered by crosstalk reaction among various battery materials or their decomposition product.^[^ ^27^, ^32^ ^]^ For instance, Liu et al. reported that the chemical crosstalk reaction between anode and cathode‐generated oxygen leads to thermal runaway in a high‐energy 25‐Ah lithium‐ion battery without short circuit.^[^ ^27^ ^]^ Huang et al. demonstrated that the exothermic reaction between electrolyte and anode‐generated LiH, the migration of H_2_ from anode to cathode attribute to inferior thermal stability of 5 Ah LiNi_0.5_Co_0.2_Mn_0.3_O_2_/graphite pouch cell.^[^ ^32^ ^]^

## 3 Nonflammable Liquid Electrolyte Design

It is essential to develop nonflammable liquid electrolyte with both improved battery safety and comparable electrochemical performance. We will discuss typical strategies on thermal and electrochemical stability design of nonflammable liquid electrolyte based on molecule, solvation, and battery level.

### 3.1 Molecular Design

**Figure 2:**
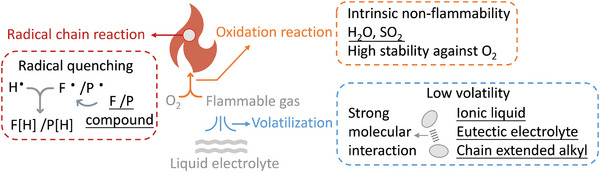
Molecular design of solvent or additive for nonflammable liquid electrolyte. Specifically, the fluorinated and phosphorus‐based compounds present high radical quenching efficiency and fire extinguishing capability. Strong molecular interaction among ionic liquid‐, eutectic, and chain extended alkyl‐based electrolyte leads to low volatility. The high stability against O_2_ of compounds, such as H_2_O and SO_2_ results intrinsic nonflammability.

The molecular structure design of nonflammable electrolyte components including solvent/additive and salt are summarized (**Tables** **1** and **2**). Specifically, radical quenching capability, intermolecular interaction, intrinsic flammability of solvent/additive (**Figure** **2**), and thermal stability of salt molecules are analyzed.

**Table 1:** Typical nonflammability electrolyte based on molecular design of solvent/additive.

Electrolyte formulation	Electrochemical performance (cell cut‐off voltage capacity retention, cycles, C‐rate)	Safety performance (performed test)
Radical quenching (fluorinated and phosphorus compounds)		
1 m LiPF_6_/EC: methyl(2,2,2‐trifluoroethyl)carbonate (FEMC) (3:7 vol)‐ 2 wt% vinylene carbonate (VC)^[^ ^39^ ^]^	Graphite||NMC622, coin cell, 4.5 V, 80%, 100 cycles, 0.5 C (45 °C)	Flame test (SET = 0 sg^−1^)
1 m LiPF_6_/PC: 2,2,2‐trifluoroethyl acetate (TFA) (3:7 vol)−2 wt% FEC^[^ ^36a^ ^]^	Graphite||NCM622, 730 mAh pouch cell, 4.3 V, 82%, 400 cycles, 1 C (45 °C)	Flame test (SET = 2.5 sg^−1^_,_ for 1 m LiPF_6_/PC: TFA (3:7 vol)
1.9 m LiFSI /2,2,2‐trifluoroethyl trifluoromethanesulfonate (TTMS):2,2,2‐trifluoroethyl methanesulfonate (TM) (1:2 vol)^[^ ^40^ ^]^	Graphite||LCO, 1 Ah pouch cell, 4.55 V, 80%, 1400 cycles Graphite||NMC811, 1 Ah pouch cell, 4.6 V, >93%, 1000 cycles 0.5 C charge, 1 C discharge	Flame test, ARC, gas expansion rate test, nail penetration
1 m LiPF_6_/methyl difluoroacetate (MDFA)/ ethoxy‐pentafluoro‐cyclotriphosphazene (PFPN)/FEC (1:7:0.5:1 mol)^[^ ^42^ ^]^	Graphite||NMC811, 240 mAh pouch cell, 4.3 V, 81.8%, 500 cycles, 1 C	Flame test (SET = 0 sg^−1^), nail penetration, volume expansion
1 m LiTFSI/ methyl difluoroacetate (MDFA): fluorosulfonyl substituted MDFA (MDFSA): 1,1,2,2‐tetrafluoroethyl‐2,2,3,3‐tetrafluoropropylether (TTE) (4:1:5 vol)^[^ ^43^ ^]^	Graphite||NMC811, pouch cell, 4.5 V, 82.8%, 360 cycles, 0.2 C (˗30 °C)	None
0.95 m LiFSI/2‐(2,2,2‐trifluoroethoxy)−1,3,2‐dioxaphospholane 2‐oxide (TFEP): 2,2,2‐trifluoroethyl methyl carbonate ((1:3 vol)^[^ ^47^ ^]^	Graphite||Li half‐cell, 0.05 V, 91.4%, 400 cycles, 0.5 C NMC||Li half‐cell, 4.5 V, 80.1%, 500 cycles, 0.5 C LNMO||Li, half‐cell, 4.9 V, 70%, 200 cycles, 0.5 C	Flame test (SET = 0 sg^−1^) Flash point (37.0 °C)
0.8 m LiTFSI/0.6 m LiDFOB/ propylene carbonate (PC): pentafluoro‐(phenoxy)‐cyclotriphosphazene (FPPN) (7:3 vol)^[^ ^36b^ ^]^	Graphite||LCO, 2.6 Ah pouch cell, 4.35 V, 80.1%, 277 cycles, 0.2 C charge, 0.5 C discharge	Flame test ARC Nail penetration Volume expansion
Molecular interaction (ionic liquid & eutectic)		
1 m 1 m LiPF_6_/EC: DMC (1:1 vol)‐ 5 mm 3‐heptyl‐1‐(3‐(3‐heptyl‐3‐phenylthioureido)propyl)−1H‐imidazole‐3‐ium hexafluorophosphate^[^ ^53^ ^]^	Graphite||NMC111, coin cell, ≈4.0 V, ≈63%, 100 cycles	Flame test
LiTFSI: succinonitrile (SCL) (1:10 mol)^[^ ^62^ ^]^	Mesocarbon microbead (MCMB)||LFP, 200 cycles, 1 C	None
LiClO_4_:Methylsulfonylmethane (MSM):H_2_O (1:1.8:1 mol)^[^ ^70^ ^]^	LTO||LMO, ≈2.8 V, 72.2%, 1000 cycles, 4.5 C	None
4.5 m LiTFSI‐0.1 m KOH/CO(NH_2_)_2_:H_2_O (8.6:1 vol)^[^ ^71^ ^]^	LTO||Li_1.5_Mn_2_O_4_, 2.5 mAh cm^−2^ pouch cell, ≈2.8 V, 92%, 470 cycles, 1 C	None
1 m LiPF_6_/EC: bis(2‐methoxyethyl) carbonate (BMEC) (3:7 vol)−3 vol% FEC^[^ ^51^ ^]^	Graphite||NMC811, 1 Ah pouch cell, ≈4.2 V, 91.4%, 500 cycles, 0.3 C	Flame test, flash point, nail penetration
Intrinsic nonflammability		
1 m Li_2_SO_4_‐0.1 m LiOH/H_2_O^[^ ^67^ ^]^	LTP||LFP, 1.4 V, 90%, 1000 cycles, 6 C	None
2 m Li_2_SO_4_^[^ ^68^ ^]^	LTP||LMO, 3–23 mAh cm^−2^ pouch cell, 1.8 V, 72%, 3000 cycles, 4 C	Dynamic bending cycles
LiAlCl_4_: SO_2_^[^ ^10a^ ^]^	Graphite||LFP, 1.08 Ah prismatic cell, ≈3.6 V, 20%, 50 000 cycles, 2 C	Flash point

**Table 2:** Thermal stability and limitations of common lithium salts.

Salt	Decomposition temperature^a)^ [°C]	Limitations
LiPF_6_	≈100^[^ ^72^ ^]^	Low thermal stability^[^ ^72^ ^]^
LiBF_4_	150–300^[^ ^72b^ ^]^	Low ionic conductivity^[^ ^74a^ ^]^
LiBOB	≈300^[^ ^74b^ ^]^	Low ionic conductivity^[^ ^74b^ ^]^
LiClO_4_	≈400–500^[^ ^72^, ^80^ ^]^	Explosive^[^ ^24^, ^75^ ^]^
LiAsF_6_	≈240^[^ ^81^ ^]^	High toxicity^[^ ^24^ ^]^
LiTFSI	≈360^[^ ^81^ ^]^	Al corrosion,^[^ ^76a^ ^]^ high cost^[^ ^77b^ ^]^
LiFSI	>200^[^ ^82^ ^]^	Al corrosion,^[^ ^76b^ ^]^ high cost^[^ ^77b^ ^]^
LiDFOB	≈240^[^ ^83^ ^]^	High cost^[^ ^77a^ ^]^

^a)^ Note: The decomposition temperature data in this table were measured using thermogravimetric analysis (TGA)/ differential scanning calorimetry (DSC), it may vary in different literature depending on experimental condition such as moisture exposure^[^ ^84^ ^]^ and solvent composition.^[^ ^85^ ^]^

#### 3.1.1 Radical Quenching

**Figure 3:**
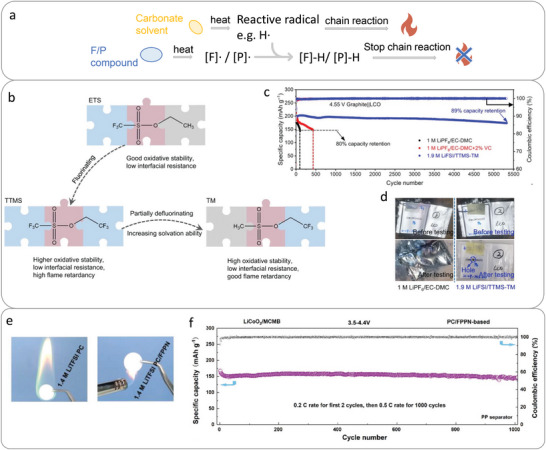
a) Mechanism and design principle of fluorinated and phosphorus‐based radical quenchers. [F] •/ [P] • present F‐/P‐containing functional groups released from fluorinated‐/phosphorus‐based flame retardant, for example, F •,^[^ ^36a^ ^]^ PO_2_ •, HPO_2_ •.^[^ ^33b^ ^]^ b) Design principle of nonflammable fluorinated solvent. c) Cycle performance of graphite||LCO cells with 1 C charge and 2 C discharge at a cut‐off voltage of 4.55 V after three pre‐cycles at 0.1 C. d) Nail penetration test of graphite||LCO cells with baseline and the designed fluorinated electrolyte. Reproduced with permission.^[^ ^40^ ^]^ Copyright 2023, Springer Nature. e) Flammability test of 1.4 m LiTFSI PC and 1.4 m LiTFSI PC/FPPN electrolyte. f) Cycle stability of MCMB||LiCoO_2_ battery using PC/FPPN‐based electrolyte. Reproduced with permission.^[^ ^36b^ ^]^ Copyright 2023, Wiley‐VCH.

Radical quenching is an effective strategy to extinguish fire by capturing flame propagating radicals including H and OH radicals, which are generated from thermal decomposition of organic solvents and responsible for the chain propagation during combustion reaction. The radical quenching chemicals dissociate upon heating and release active radical species to scavenge the H•/OH• radicals (as shown in Figures 2 and 3a, leading to an interrupted combustion reaction and improved safety.^[^ ^33^ ^]^ The common radical quenching chemicals are fluorinate‐based and phosphorous‐based organics,^[^ ^12^, ^34^ ^]^ they are used as additive (<10%,^[^ ^35^ ^]^ in weight or volume ratio), cosolvent (10%–100%.^[^ ^36^ ^]^), or single solvent (100%) for nonflammable liquid electrolyte in Li‐ion batteries. Other radical quenching materials also demonstrated an effective flame retardancy, for example, Zhang et al. reported that a nonflammable amide (dimethylacetamide, DMAC) could generate NCO• radical to scavenge H•/OH• radicals because the binding energy between dimethylacetamide (DMAC) and H•(−0.12 kJ mol^−1^)/OH•(−0.12 kJ mol^−1^) is lower than that between DMC and H•(−0.04 kJ mol^−1^)/OH•(−0.77 kJ mol^−1^).^[^ ^37^ ^]^ However, it is reported that the electrolyte of sodium bis(oxalato)borate (NaBOB, 0.70 molarity) in DMAC could be flammable.^[^ ^38^ ^]^ Perhaps the flame retardancy of DMAC is influenced by the salt (NaBOB). The flammability test results may also differ owing to lack of unified ignition conditions, such as flame temperature and ignition time. Further investigation is required to evaluate and design the efficiency of different radical quenching materials. Herein, we discuss more details about fluorinate‐ and phosphorous‐based chemicals which have been extensively studied.

##### Fluorinated Compounds

Fluorinated compounds including carbonate (methyl(2,2,2‐trifluoroethyl)carbonate),^[^ ^39^ ^]^ ester (2,2,2‐trifluoroethyl acetate),^[^ ^36a^ ^]^ sulfonate (2,2,2‐trifluoroethyl trifluoromethanesulfonate)^[^ ^40^ ^]^), and alkanes (liquefied gas 1,1,1,2‐tetrafluoroethane, pentafluoroethane)^[^ ^41^ ^]^ can terminate fire effectively by producing fluorine radicals (F•) and capturing reactive radicals (for example, H•).^[^ ^36a^ ^]^ The higher bond strength of C*─*F (105.4 kcal mol^−1^) compared with that of C*─*H (98.8 kcal mol^−1^) also contributes to a higher thermal stability of fluorinated components.^[^ ^31^ ^]^ In addition, the fluorinated electrolytes present advantages in electrochemical performance including: 1) The high electron‐withdrawing effect of fluorine atoms resulted from its high electronegativity improves anodic stability of electrolyte and enables high‐voltage Li‐ion batteries.^[^ ^42^ ^]^ 2) Formation of a robust LiF‐rich SEI or CEI from fluorinated electrolyte improves the cycle performance of batteries.^[^ ^40^ ^]^ 3) Fluorinated compounds with a low freezing point, soft Li^+^ solvating ability, and moderate boiling point supports a wide temperature operation of Li‐ion battery.^[^ ^43^ ^]^ For example, Zhang et al.^[^ ^40^ ^]^ designed a nonflammable electrolyte with 1.9 m LiFSI in a mixture of 2,2,2‐trifluoroethyl trifluoromethanesulfonate (TTMS) and 2,2,2‐trifluoroethyl methanesulfonate (TM) (1:2 v/v), which benefits the formation of efficient SEI on graphite anode and CEI on high‐voltage cathodes, enabling a good cycling performance of 4.55 V graphite||LiCoO_2_ battery with a 89% capacity retention over 5329 cycles, and 4.6 V graphite||NCM811 battery with a 85% capacity retention over 2002 cycles (**Figure** **3**b–d). Xu et al.^[^ ^43^ ^]^ reported that a 4.5 V graphite||NCM811 can operate effectively at a temperature range from −60 to +60 °C with a nonflammable fluorinated electrolyte, which consists of methyl difluoroacetate (MDFA), MDFSA (fluorosulfonyl substituted MDFA), and 1,1,2,2‐tetrafluoroethyl‐2,2,3,3‐tetrafluoropropylether (TTE) with 1 m LiTFSI salt.

However, fluorine‐containing compounds also raise concerns of cost and toxicity.^[^ ^33b^ ^]^ Therefore, the molecular design of a lower content of fluorine is required to balance the safety, cost, and performance of Li‐ion batteries.

##### Phosphorus Compounds

Phosphorus‐based compounds present high flame‐retarding ability owing to its ability to capture H radical,^[^ ^44^ ^]^ and they are more environmentally friendly compared to fluorine‐based compound.^[^ ^15^ ^]^ Specifically, phosphates with a low molecular mass including trimethyl phosphate (TMP) and triethyl phosphate (TEP) are considered as popular candidates owing to their low viscosity, good Li salt solubility, high oxidative stability, and wide operating temperature range.^[^ ^45^ ^]^

However, phosphorus‐based compounds are not compatible with graphite anode owing to the weak SEI formation.^[^ ^46^ ^]^ For example, the cycling performance fades notably when the concentration of TMP exceeds 10 wt% in a baseline electrolyte (1.0 m LiPF_6_ in EC and EMC with vinylene carbonate (VC) as additive).^[^ ^46^ ^]^

Molecular modifications have been proposed to improve SEI quality of phosphorus molecule: 1) partial fluorination;^[^ ^36^, ^47^ ^]^ 2) increasing polymerizing ability.^[^ ^48^ ^]^ Zheng et al.^[^ ^47^ ^]^ designed a fluorinated cyclic phosphate solvent (2‐(2,2,2‐trifluoroethoxy)−1,3,2‐dioxaphospholane 2‐oxide, TFEP) which combines the advantages of fluorination and cyclic phosphate structure to generate an effective SEI. The reported nonflammable electrolyte of 0.95 m LiN(SO_2_F)_2_ in TFEP/2,2,2‐trifluoroethyl methyl carbonate (1:3 v/v) shows a stable cycle performance of graphite anode and LiNi_0.5_Mn_1.5_O_4_ cathode. Lu et al.^[^ ^36b^ ^]^ employed pentafluoro(phenoxy)cyclotriphosphazene (FPPN) as noncoordinating flame‐retardant to adjust solvation shell structure of Li^+^ and modify SEI layer of graphite. A high‐capacity retention of 91.2% over 1000 cycles at 0.5 C is achieved for 4.4 V mesocarbon microbeads (MCMB)||LiCoO_2_ battery with the proposed 1.4 m LiTFSI PC/FPPN (7:3, by volume) electrolyte (Figure 3e,f).

#### 3.1.2 Intermolecular Interaction

Volatility and flash point of electrolyte is associated with intermolecular interaction among electrolyte molecules.^[^ ^49^ ^]^ Ionic liquid and eutectic solutions have been widely reported to construct nonflammable electrolyte owing to their strong intermolecular interaction and low volatility.^[^ ^50^ ^]^ In addition, molecular regulation of organic carbonate chain is effective to increase flash point and lower electrolyte flammability.^[^ ^51^ ^]^

##### Ionic Liquid

**Figure 4:**
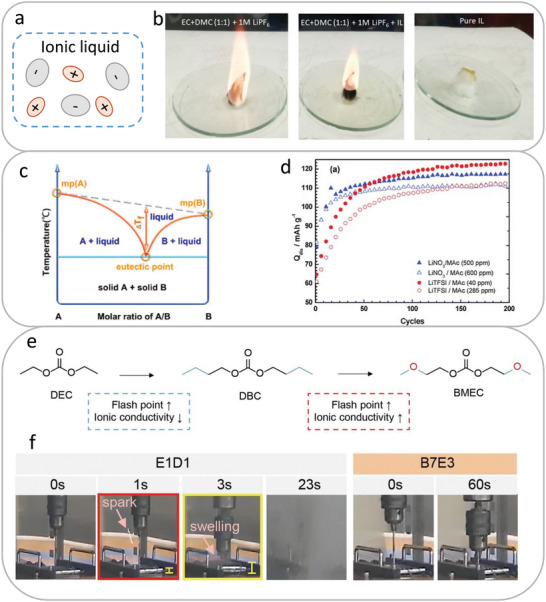
a) Schematic illustration of ionic liquid electrolyte. b) Comparison of flammability of the commercial electrolyte (left), the commercial electrolyte with ionic liquid (IL) (middle), and the pure IL (right). Reproduced with permission.^[^ ^53^ ^]^ Copyright 2020, American Chemical Society. c) Phase diagram of eutectic electrolyte which consists of two components. Reproduced with permission.^[^ ^58^ ^]^ Copyright 2022, American Chemical Society. d) Cycle performance of LFP using LiTFSI‐*N*‐methylacetamide (MAc) or LiNO_3_‐MAc electrolyte at 1 C. Reproduced with permission.^[^ ^60^ ^]^ Copyright 2013, Royal Society of Chemistry. e) Design principle of nonflammable organic carbonate BMEC. f) Photos of nail penetration of graphite||NMC811 pouch cells using 1 m LiPF_6_ with 3 vol% FEC in EC/DEC (1:1, v/v) (E1D1) and 1 m LiPF_6_ with 3 vol% FEC in BMEC/EC (7:3, v/v) (B7E3) electrolytes. Reproduced with permission.^[^ ^51^ ^]^ Copyright 2013, Royal Society of Chemistry.

Ionic liquid consists of organic or inorganic–organic hybrid salts with cations including ammonium, sulfonium, imidazolium (C*_n_* MIm), and anions such as FSI^−^, TFSI^−^, and BF^4−[^ ^12^, ^50^ ^]^ (**Figure** **4**a). They are in liquid form at room temperature.^[^ ^52^ ^]^ Ionic liquid has been commonly used for its wide electrochemical window, adjustable viscosity, high thermal stability, negligible vapor pressure, and low flammability.^[^ ^53^ ^]^ The low flammability of ionic liquid results from its low volatility that associated with strong intermolecular forces between ionic liquid ions.^[^ ^50a^ ^]^ It is reported that the addition of a small amount (5 mm) of ionic liquid 3‐heptyl‐1‐(3‐(3‐heptyl‐3‐phenylthioureido)propyl)−1H‐imidazole‐3‐ium hexafluorophosphate to the commercial Li‐ion electrolyte delays the ignition and enhances the safety of Li‐ion batteries (Figure 4b).^[^ ^53^ ^]^

However, the usage of ionic liquid causes issues of low ionic conductivity,^[^ ^54^ ^]^ high cost, eco‐toxicity,^[^ ^55^ ^]^ and corrosion of Al current collector especially when using TFSI or FSI anions which lacks the capability to generate a LiF protection film for Al.^[^ ^56^ ^]^ Furthermore, thermal decomposition of some ionic liquid results in ignitable gases, leading to combustion reaction in the batteries.^[^ ^57^ ^]^ Therefore, more comprehensive investigation and optimization is needed for the application of ionic liquid for nonflammable Li‐ion electrolyte.

##### Eutectic Electrolyte

Eutectic electrolyte is composed of two or more components, the strong intermolecular forces including hydrogen‐bond, Lewis acid‐base, and van der Waals interactions between different components results in a lower melting point compared with that of individual component (Figure 4c).^[^ ^50b^ ^]^ It shares some similar features of ionic liquid such as low volatility, high thermal stability, and wide electrochemical window. Furthermore, eutectic electrolyte shows a more facile preparation, lower cost, and toxicity compared with ionic liquids, and it has arisen a wide research attention in the field Li‐ion battery.^[^ ^50^, ^58^ ^]^ For instance, the mixture of Li salt (such as LiTFSI, LiPF_6_, LiClO_4_, LiFSI) and solvent (such as urea,^[^ ^59^ ^]^ *N*‐methylacetamide (NMAc),^[^ ^60^ ^]^ acetamide,^[^ ^61^ ^]^ succinonitrile^[^ ^62^ ^]^) have been reported for Li‐ion battery. Specifically, the amide‐type chemicals (urea, NMAc, acetamide, etc.) have been widely used as effective hydrogen bond donor species to build eutectic electrolyte owing to their N*─*H bonds.^[^ ^63^ ^]^ For example, Boisset et al. demonstrated a good compatibility between NMAc‐based eutectic electrolyte and LFP electrode with a stable cycle performance over 200 cycles (Figure 4d).^[^ ^60^ ^]^

However, deeper investigation of coordination mechanism and material optimization of eutectic electrolyte is required to improve its ionic conductivity and cathodic stability.^[^ ^58^ ^]^ For instance, the N*─*H bonds in amides suffer from a low stability against reduction,^[^ ^63^ ^]^ modification on chemical structure or passivation on anode surface are suggested to suppress the decomposition of amides. Furthermore, multifunctional additive which increases ionic conductivity and SEI quality is a promising strategy for improving the performance of eutectic electrolyte. For example, fluoroethylene carbonate (FEC) has been reported to simultaneously build a robust SEI and improve ionic conductivity for LiTFSI‐NMAc based eutectic electrolyte.^[^ ^64^ ^]^

##### Organic Carbonate

Flammability of organic carbonate‐based electrolyte can be lowered by increasing intermolecular interaction of carbonate molecules. Lee et al.^[^ ^51^ ^]^ reported that extending the alkyl chain of DEC molecule with *─*C_2_H_5_ results in a stronger intermolecular interaction and higher vaporization enthalpy, leading to a decreased vapor pressure, higher flash point (from 31 to 121 °C) and lower flammability. To further increase the polarity of the solvent and improve the ion conductivity, additional ethereal oxygen was introduced to obtain bis(2‐methoxyethyl) carbonate (BMEC)) molecule (Figure 4e). The designed nonflammable electrolyte 1 m LiPF_6_ in BMEC/EC (7:3 v/v) enables 91.4% capacity retention of 1 A h graphite||NMC811 battery over 500 cycles and successfully prevents the thermal runaway of a 4 A h graphite||NMC811 pouch cell during nail‐penetration test (Figure 4f).

#### 3.1.3 Intrinsic Nonflammability

Combustion reaction is intrinsically an oxidation reaction of ignition source with released heat.^[^ ^65^ ^]^ A high stability against oxygen (such as H_2_O, SO_2_^[^ ^12b^ ^]^) results in an intrinsic nonflammability.

Extensive attention has been paid to develop water‐based electrolyte.^[^ ^10^, ^66^ ^]^ In addition to nonflammability, aqueous electrolyte also presents advantages of 1) a higher ionic conductivity which benefits fast charging of batteries; 2) a lower cost of material and manufacturing compared with that of traditional nonaqueous electrolyte.^[^ ^66^ ^]^ To further improve cycle stability, several strategies such as pH adjustment and oxygen elimination of aqueous electrolyte,^[^ ^67^ ^]^ electrode coating, and 3D electrode architecture^[^ ^68^ ^]^ have been applied. However, the stability window of traditional aqueous electrolyte is low (<2.0 V) due to water decomposition, leading to a limited energy density of aqueous battery.^[^ ^69^ ^]^ Therefore, it is critical to improve water stability for the practical application of aqueous Li‐ion battery.

SO_2_‐based liquified gas electrolyte has also been reported as a nonflammable electrolyte for Li‐ion batteries. A 1.08 Ah graphite||LFP Li‐ion battery with LiAlCl_4_ dissolved in SO_2_ was demonstrated with a capacity retention of 20% after 50 000 cycles at 2 C.^[^ ^10a^ ^]^ Although it presents a low flammability and high ionic conductivity (121 mS cm^−1^ at 22 °C), it is highly volatile and prone to corrode current collector,^[^ ^12a^ ^]^ limiting its practical application.

#### 3.1.4 Salt Design

LiPF_6_ is a commercially used salt owing to its balanced properties of ionic conductivity, stability toward Al, cost, and toxicity.^[^ ^24^ ^]^ However, it decomposes to form LiF and PF_5_ at a low temperature of ≈100 °C,^[^ ^72^ ^]^ and the strong Lweis PF_5_ will react with solvent (e.g., EC),^[^ ^73^ ^]^ inducing more thermal decompositions within battery. Table 2 shows typical Li salts which are more thermally stable than LiPF_6_, they present different thermal behaviors based on their chemical structure. For instance, it is reported that the decomposition of LiBF_4_ salt is endothermic because a large energy is required to break its chemical bond, while the oxygen‐containing salts, such as LiClO_4_ and lithium bis(trifluoromethanesulfonyl)imide (LiC_2_NO_4_F_6_S_2_, LiTFSI) release heat owing to the exothermic oxidation reaction during their decomposition process.^[^ ^72b^ ^]^ In terms of influence on SEI, the LiBF_4_‐containing electrolyte generates a thermally unstable SEI which is mainly composed of LiF and lithium alkyl carbonate and decomposed at around 60 °C, while the SEI from LiTFSI‐, lithium bis(oxalato)borate (LiBOB)‐, and lithium difluoro(oxalate) borate (LiDFOB)‐based electrolytes which consists of Li*_x_* SO*_y_*, Li_2_S, Li_3_N, LiF, B_2_O_3_ shows a higher thermal stability with a onset of SEI decomposition at 110–160 °C.^[^ ^73^ ^]^ More investigation is needed to provide a comprehensive understanding of influence of different salts on battery safety.

Although those salts have demonstrated an improved thermal stability in some perspectives, such as decomposition temperature and thermal stability of SEI, several key issues are limiting their practical application. For example, the ionic conductivity of LiBF_4_ and LiBOB is low at room temperature.^[^ ^74^ ^]^ LiClO_4_ is explosive owing to its high oxidation ability and is usually limited in lab scale.^[^ ^24^, ^75^ ^]^ The application of LiAsF_6_ is limited due to its highly poisonous reductive product As(0) and As(III) species.^[^ ^24^ ^]^ LiTFSI and lithium bis(fluorosulfonyl)imide (LiFSI) are prone to cause Al corrosion at a low concentration.^[^ ^76^ ^]^ In addition, LiTFSI, LiFSI, and LiDFOB suffer from high cost.^[^ ^77^ ^]^

Blended‐salt electrolyte which consists of different salts has been proposed to achieve both improved thermal stability and good cell performance of Li‐ion battery.^[^ ^78^ ^]^ For instance, the electrolyte of 0.8 m LiTFSI‐0.2 m LiDFOB‐0.01 m LiPF_8_ in EC:PC (1:1 by volume) shows a higher thermal stability (52 °C higher in onset temperature of thermal runaway) and better cycling performance than that of 1 m LiPF_6_ in EC:DMC (1:1 by volume) electrolyte,^[^ ^79^ ^]^ suggesting the importance of designing effective blended‐salt electrolyte to improve the safety and performance of Li‐ion battery.

### 3.2 Solvation Structure Design

**Figure 5:**
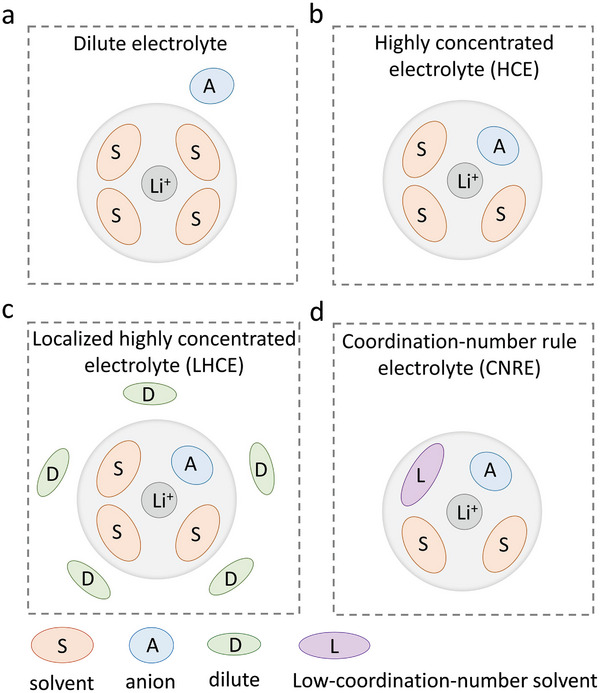
Schematic illustrations of solvation structure of a) traditional dilute electrolyte, b) highly concentrated electrolyte, c) localized highly concentrated electrolyte, and d) coordination‐number rule electrolyte. Note that the solvation number per lithium cation varies in different conditions such as salt concentration,^[^ ^86^ ^]^ and molar mass of solvent.^[^ ^87^ ^]^ Here the case of four is shown for illustration.

The regulation of solvation structure with salt and solvent/additive has been reported as an effective method to construct nonflammable electrolyte with improved safety and cycle stability. Typically, they can be categorized as highly concentrated (HCE), localized highly concentrated (LHCE), and coordination‐number rule electrolyte (CNRE), as shown in **Figure** **5** and **Table** **3**.

**Table 3:** Typical nonflammable electrolyte based on solvation design.

Electrolyte formulation	Electrochemical performance (cell cut‐off voltage capacity retention, cycles, C‐rate) *	Safety performance (performed test)
Highly concentrated electrolyte (HCE)		
LiFSI: DMC (1: 1.1 mol)^[^ ^89^ ^]^	Graphite||LNMO, 4.8 V, 90%, 100 cycles, 0.2 C (40 °C)	Flame test
5.3 m LiFSI/TMP^[^ ^45b^ ^]^	Graphite||LNMO, 4.8 V, 100 cycles, 0.2 C	Flame test, flash point
21 m LiTFSI/H_2_O^[^ ^69a^ ^]^	Mo_6_S_8_||LMO, coin cell, ≈2.3 V, 68%, 1000 cycles, 4.5 C 78%, 100 cycles, 0.15 C	None
Li(TFSI)_0.7_(BETI)_0.3_•2H_2_O^[^ ^100^ ^]^	LTO||LCO, ≈2.8 V, 75%, 200 cycles, 10 C LTO||LNMO, ≈3.4 V, 63%, 100 cycles, 6.8 C	None
Localized highly concentrated electrolyte (LHCE)		
LiFSI: DME: FEC: ethoxy(pentafluoro) cyclotriphosphazene (PFPN) (1:1.5:0.5:3 mol)^[^ ^94^ ^]^	Graphite||NMC811, coin cell, 4.5 V, 82%, 1000 cycles, 1/3 C 4.6 V, 89.8%, 300 cycles, 1/3 C	Flame test
1.44 m LiFSI/ TMP: FEC: 1,1,2,2‐tetrafluoroethyl 2,2,3,3‐tetrafluoropropyl ether (TTE) (1.2:0.2:3 mol)^[^ ^46^ ^]^	Graphite||NMC811, coin cell, 4.4 V, 85.4%, 500 cycles Charge C/3, discharge 1 C	Flame test
12.5 m LiNO_3_ 1,5‐H_2_O: pentanediol (PD) (1:1 mass)^[^ ^95^ ^]^	Mo_6_S_8_||LMO, ≈2.3 V, 250 cycles, 1 C (with gel)	Flame test
Coordination number rule electrolyte (CNRE)		
1 m LiPF_6_/tris(2,2,2‐trifluoroethyl) phosphate (TFEP): fluoroethylene carbonate (FEMC) (1:1.25 mol)^[^ ^86b^ ^]^	Graphite||NMC811, 5 Ah pouch cell, 4.3 V, 82.7%, 250 cycles, 0.2 C	Flame test, nail penetration

#### 3.2.1 Highly Concentrated Electrolyte (HCE)

**Figure 6:**
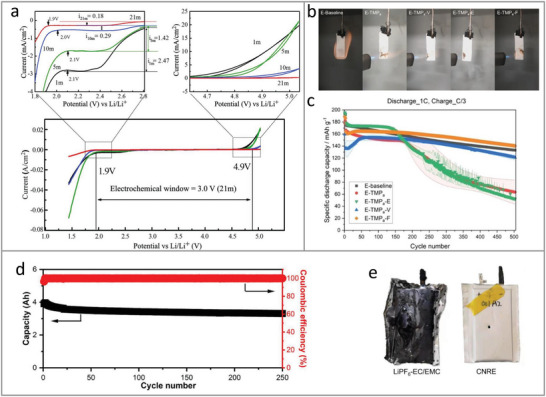
Solvation design of nonflammable electrolytes based on a) high concentration, b,c) localized high concentration, and d,e) coordination number rule. a) The stability window of aqueous electrolyte with 1, 5, 10, and 21 m LiTFSI. Reproduced with permission.^[^ ^69a^ ^]^ Copyright 2015, Science. b) Flammability test of TMP‐based locally high concentration electrolyte (LHCE). c) Cycle performance of graphite||NMC811 with TMP‐based LHCE. Reproduced with permission.^[^ ^46^ ^]^ Copyright 2021, Wiley‐VCH. d) Stability test of graphite||NMC using coordination number rule electrolyte (CNRE). e) Image of graphite||NMC pouch cell after nail penetration test. Reproduced with permission.^[^ ^86b^ ^]^ Copyright 2023, Elsevier.

The concept of high‐concentration “solvent‐in‐salt” electrolyte can be traced back to 1993, describing that the ratio of salt is higher than that of solvent, which is reversed to traditional dilute electrolyte.^[^ ^88^ ^]^ The high‐concentration structure contributes to 1) a decreased flammability owing to a lower content of flammable solvent;^[^ ^89^ ^]^ 2) an improved stability against Al corrosion when using some thermal stable salts which suffer from Al corrosion (e.g., LiTFSI,^[^ ^90^ ^]^ LiFSI^[^ ^89^ ^]^); 3) a higher electrochemical stability due to a higher content of anion in the Li solvation shell (Figure 5b), which leads to a more robust SEI.^[^ ^45^, ^69^ ^]^ For instance, concentrated electrolyte of LiFSI in DMC (1:1.1 in molar ratio) presents a lower flammability and suppressed Al corrosion, leading to an improved safety and cycle performance (90% capacity retention over 100 cycles) of graphite||LiNi_0.5_Mn_1.5_O_4_ battery.^[^ ^89^ ^]^ Concentrated 5.3 m LiFSI in TMP^[^ ^45b^ ^]^ enables the stable cycle of Li‐ion batteries owing to the protection of a passivating SEI and suppressed decomposition of phosphorus‐based solvent. Suo et al.^[^ ^69a^ ^]^ proposed 21 m LiTFSI “water‐in‐salt” electrolyte and increase the stability window of aqueous Li‐ion batteries from 2.0 to 3.0 V with the formation of effective SEI (**Figure** **6**a). Based on the development of “water‐in‐salt” electrolyte, the strategies of regulating water hydrogen bond such as LiTFSI‐H_2_O‐methylurea (MU)_0.27_,^[^ ^91^ ^]^ and 2 m LiTFSI‐94% polyethylene glycol (PEG)−6% H_2_O^[^ ^92^ ^]^ were proposed to further improve cycle stability, or reduce salt usage which associated with the issues of cost and toxicity.^[^ ^13^, ^93^ ^]^ In addition to the requirement of a large content of Li salt, the high viscosity, poor ionic conductivity, and wettability of concentrated electrolytes also limits their practical application.^[^ ^93^ ^]^

#### 3.2.2 Localized Highly Concentrated Electrolyte (LHCE)

LHCE is an effective strategy to lower cost and viscosity in high‐concentration electrolyte. The content of salt is reduced by adding a diluent which barely coordinates with Li, leading to a similar solvation structure of high‐concentration electrolyte (Figure 5c). For instance, Chen et al.^[^ ^94^ ^]^ used flame‐retarding ethoxy(pentafluoro) cyclotriphosphazene (PFPN) as a nonsolvating agent to dilute concentrated LiFSI electrolyte, the graphite||NMC811 battery with this nonflammable LHCE presents a high capacity retention of 82.0% over 1000 cycles at 4.5 V and 89.8% over 300 cycles at 4.6 V. The TMP‐based LHCE was reported with 1,1,2,2‐tetrafluoroethyl 2,2,3,3‐tetrafluoropropyl ether (TTE) as a dilute.^[^ ^46^ ^]^ The designed low‐flammable electrolyte 1.44 m LiFSI in TMP‐FEC‐TTE (1.2:0.2:3.0 by molar ratio) enables 4.4 V graphite||NMC811 cells with a capacity retention of 85.4% over 500 cycles (Figure 6b,c). Jaumaux et al.^[^ ^95^ ^]^ reported a LHCE aqueous electrolyte (12.5 m LiNO_3_ in H_2_O: 1,5‐pentanediol (PD) (1:1 by mass)) by using PD as a diluent, achieving a similar electrochemical stability window (≈2.9 V) as that of concentrated aqueous electrolyte (e.g., 21 m LiTFSI) at a lower concentration of cheaper Li salt (12.5 m LiNO_3_).

Noted that although diluent weakly solvates with Li^+^ ion and almost preserves the solvation structure of HCE,^[^ ^96^ ^]^ it could still affect the coordination environment of Li^+^ ion. For instance, van Ekeren et al. reported that the diluents bis(2,2,2‐trifluoroethyl) ether (BTFE) and TTE have influence on the Li^+^‐TEP solvation structure, namely the electron density around Li^+^ decreases with the addition of BTFE and TTE in LHCE.^[^ ^97^ ^]^ To gain a deeper understanding of solvation structure of LHCE, comprehensive investigation, such as the combination of Raman and nuclear magnetic resonance (NMR) spectroscopy, as well as the study of a broader range of the spectrum is suggested for a more accurate analysis.

However, most diluent is a highly fluorinated compound, which is expensive, toxic, and even flammable.^[^ ^12b^ ^]^ Therefore, it is critical to develop effective dilute with a lower F content or even nonfluorinated structure.^[^ ^98^ ^]^ In addition, the ionic conductivity of LHCE is still not high enough especially for the application in low temperature environment owing to the low dissociation degree of Li salt in LHCE.^[^ ^86b^ ^]^

#### 3.2.3 Coordination Number Rule Electrolyte (CNRE)

The coordination number rule was proposed to regulate solvation structure by introducing low‐coordination‐number solvents into high‐coordination‐number solvent electrolyte.^[^ ^86^, ^99^ ^]^ The addition of low‐coordination‐number solvents results in an unsaturated coordination of solvent molecules around Li^+^, enabling the participation of anion in the first solvation shell of Li and leading to a higher cathodic stability of solvent molecules and a more robust SEI.^[^ ^86^, ^99^ ^]^ Radical quenching phosphorus‐based electrolyte suffers from a low cathodic stability owing to the high coordination number of phosphorus compounds (e.g., TMP,^[^ ^99^ ^]^ tris(2,2,2‐trifluoroethyl) phosphate (TFEP)^[^ ^86b^ ^]^). In order to improve electrochemical stability of nonflammable phosphorus‐based electrolyte, the low‐coordination‐number fluoroethylene carbonate (FEMC) was employed to construct 1 m LiPF_6_/tris(2,2,2‐trifluoroethyl) phosphate (TFEP): fluoroethylene carbonate (FEMC) (1:1.25 mol) CNRE electrolyte.^[^ ^86b^ ^]^ A high cycle stability (82.7% capacity retention after 250 cycles) and safety (verified by nail penetration test) was achieved for graphite||NMC811 pouch cell with the designed CNRE electrolyte (Figure 6d,e). In addition, a high ionic conductivity especially at a low temperature was achieved for this CNRE electrolyte compared to that of HCE and LHCE electrolyte due to the altered solvation structure and dissociation degree of lithium salt.^[^ ^86b^ ^]^

### 3.3 Cell Compatibility Design

**Figure 7:**
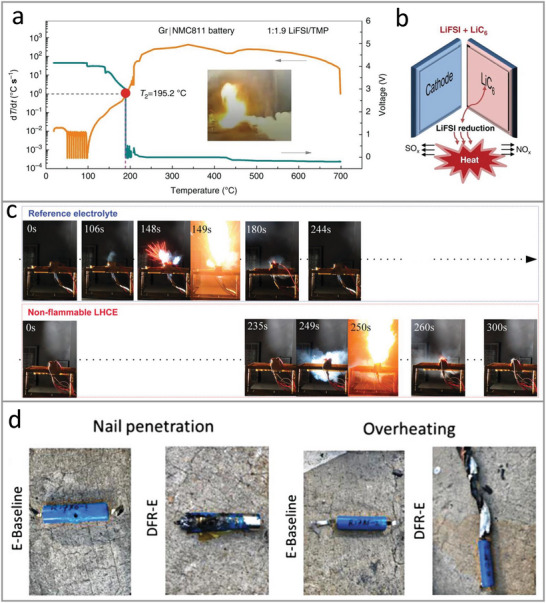
Cell compatibility investigation of nonflammable liquid electrolyte. a) The temperature measurement of d*T*/d*t* of the graphite||NMC811 cell using the nonflammable concentrated LiFSI∖TMP electrolyte. b) Schematic illustration of thermal runaway mechanism in graphite||NMC811 battery with the concentrated LiFSI/TMP, indicating that the heat produced from the reaction between LiFSI and charged graphite leads to battery thermal runaway. Reproduced with permission.^[^ ^101^ ^]^ Copyright 2020, Springer Nature. c) Comparison of charged graphite||NMC811 pouch cell with reference and nonflammable electrolytes during thermal runaway. Reproduced with permission.^[^ ^102^ ^]^ Copyright 2022, Wiley‐VCH. d) Photographs of graphite||LFP cells before and after nail penetration and overheating. The DFR‐E is 2.6 m LiFSI in TMP‐EC‐TTFEPi (1:0.09:1.67 by mass) electrolyte, the E‐baseline is 1 m LiPF_6_ in EC: EMC (3:7 by mass) with 2 wt% vinylene carbonate (VC). Reproduced with permission.^[^ ^14^ ^]^ Copyright 2022, Wiley‐VCH.

Reducing electrolyte flammability is a critical, but not ultimate solution to battery safety. The investigation and design of safety compatibility between electrolyte and other battery components is also essential to build a safe battery for practical applications. Several reports demonstrate that fire hazard can still exist even with a nonflammable electrolyte. For example, Hou et al.^[^ ^101^ ^]^ reported that the exothermic reaction between LiFSI and the lithiated graphite occurs when using nonflammable concentrated LiFSI/TMP (1:1.9 by molar) electrolyte, leading to fire issues of graphite||NMC811 battery (**Figure** **7**a,b). The graphite‐SiO||NMC811 pouch cell with nonflammable LHCE ((1.0 m LiFSI in FEC:TEP: bis(2,2,2‐trifluoroethyl) ether (BTFE) (1:2:7 by volume)) also suffers from combustion (Figure 7c).^[^ ^102^ ^]^ Jia et al.^[^ ^14^ ^]^ investigated the safety of 1.2 Ah graphite||LFP battery with completely nonflammable 2.6 m LiFSI in TMP‐EC‐TTFEPi (1:0.09:1.67 by mass) electrolyte, and concluded that the reactions between LiFSI and both charged cathode and anode lead to thermal runaway of battery (Figure 7d), which is even more severe than the battery using baseline electrolyte (1 m LiPF_6_ in EC:EMC (3:7 by mass) with 2 wt% vinylene carbonate (VC)). The reported high reactivity between LiFSI and charged electrodes indicates that a further optimization of LiFSI‐containing electrolyte is required for battery safety even though it is already nonflammable.

In addition to LiFSI‐containing electrolytes, comprehensive investigations on the exothermic reaction between other nonflammable electrolytes (for example, LiTFSI and water‐containing electrolytes) and cell components (such as charged electrodes, current collector) are needed to design safe Li‐ion battery. Three strategies are proposed to suppress the reaction between electrolyte and various cell components: 1) Alter the chemical structure of electrolyte molecules to improve its chemical stability at a high temperature; 2) Build a protective layer on the surface of other cell components to improve their thermal stability; 3) Design thermo‐responsive additive to deactivate electrolyte components or charged electrode to mitigate thermal runaway reaction upon high temperature.

## 4 Safety Test

**Figure 8:**
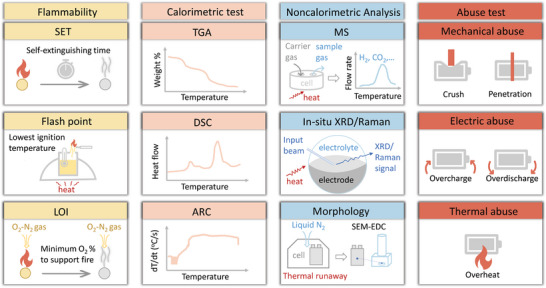
Schematic illustration of safety test for Li‐ion batteries.

Comprehensive safety test is crucial to deepen mechanistic understanding of thermal runaway and provide reliable evaluation for practical application. Typical safety tests of thermal stability include flammability test (electrolyte level), calorimetric/noncalorimetric characterization (material or battery level), and abuse test (battery level) are discussed (**Figure** **8**).

### 4.1 Flammability Test

Qualitative ignition experiment is a commonly used method to evaluate the flammability of electrolytes by observing whether fire can be extinguished with target electrolyte.^[^ ^14^, ^92^, ^102^ ^]^ The testing conditions differs in many aspects, such as the flame temperature, ignition time, and the material of the targeted electrolyte soaked or contained in (e.g., Celgard, glass fiber, or coin cell shell). Commercial electrolyte is usually selected as a comparison to highlight the low flammability of designed electrolyte.

Quantitative evaluation of electrolyte flammability includes the measurement of self‐extinguishing time (SET), flash point, and limiting oxygen index (LOI).

SET represents the time that a sample burns after ignition.^[^ ^103^ ^]^ The majority of reported SET experiments of electrolytes measured the extinguishing time of porous solid materials (such as glass fiber) which immobilizing with liquid electrolytes. Xu et al.^[^ ^104^ ^]^ proposed the usage of small ball‐shaped wicks soaked with 0.05–0.1 g electrolyte to minimize electrolyte exposure to air and avoid the influence of wettability. They also classified the electrolytes into three types: 1) nonflammable if SET < 6 s g^−1^; 2) flammable if SET > 20 s g^−1^; 3) flame‐retarded if 6 s g^−1^ <SET<20 s g^−1^.

Flash point is the lowest temperature when the vapor is ignited with an flame source.^[^ ^103^ ^]^ The liquids can be classified as flammable or combustible depending on flash point. According to Occupational Safety and Health Administration (OSHA) and National Fire Protection Agency (NFPA), the liquid is defined as flammable if flash point <38 °C, and combustible when flash point is between 38 and 93 °C.^[^ ^105^ ^]^

Limiting oxygen index (LOI) indicates the minimum oxygen content that is required for the combustion of a sample.^[^ ^106^ ^]^ It is measured by passing a mixture gas of N_2_ and O_2_ to a burning material. The content of O_2_ keeps reducing until the material stops burning, and the obtained critical O_2_ content is considered as LOI.^[^ ^106^ ^]^ The flammability of liquid can be categorized into 1) flammable when LOI <21%; 2) retarded if 21%< LOI <28%; 3) nonflammable for LOI >28%.^[^ ^107^ ^]^

### 4.2 Calorimetric Test

Calorimetric techniques including thermogravimetric analysis (TGA), differential scanning calorimetry (DSC), and accelerated‐rate calorimetry (ARC) have been widely used to investigate the thermal properties and heat generation of Li‐ion battery components.^[^ ^108^ ^]^

TGA shows the weight changes of a sample when the temperature is increasing, providing information for decomposition mechanism of target system upon heating.^[^ ^106^ ^]^ It has been adopted to study thermal stability of electrolyte. For instance, TGA results of 1 m LiPF_6_ in the solvent of EC/EMC and nonflammable vinylethylene carbonate (VEC) shows that the weight loss of VEC‐based electrolyte (≈18%) is smaller than that of EC/EMC‐based (≈42%), indicating a lower volatility and potential higher thermal safety of VEC.^[^ ^109^ ^]^

DSC measures the change of heat needed for a reference and test sample when they are subjected to a temperature program.^[^ ^106^ ^]^ This technology has been employed for thermal analysis of partial cell components and full cell components.^[^ ^101^, ^110^ ^]^ The temperature onset of a exothermic peak and calculated heat value can be used to compare the thermal stability of different test system.^[^ ^101^ ^]^

ARC is a powerful technology to study thermal behavior of battery materials, single battery and battery packs at an adiabatic condition.^[^ ^111^ ^]^ Several critical features of ARC curve have been widely evaluated for investigating thermal safety of Li‐ion batteries such as self‐heating rate (d*T*/d*t*), three characteristic temperatures including onset temperature of self‐heating T1, onset temperature of thermal runaway T2, and maximum temperature during thermal runaway T3.^[^ ^101^, ^102^ ^]^ ARC test plays a key role in providing deep understanding of thermal safety of batteries.

### 4.3 Noncalorimetric Characterization

Characterization of noncalorimetric features of battery materials including structure evolution and gas generation can be in situ performed during heating or combined with calorimetric test for thermal runaway investigation. For example, in situ high‐energy X‐ray diffraction (HEXRD) has been reported to detect the chemical reactions between electrolyte and NMC materials in high temperature.^[^ ^112^ ^]^ In situ Raman test was conducted to study the thermal stability of near‐surface structure of NCM materials with different electrolytes, which are sealed in a temperature control device.^[^ ^113^ ^]^ Mass spectrometry (MS) can provide information of real‐time gas analysis during a reaction,^[^ ^114^ ^]^ it has been extensively used to analyze the gas evolution of battery in high temperature.^[^ ^22^, ^115^ ^]^ The result of MS combined with DSC shows that the generation of several reductive gases (for example, alkene, alkynes) from electrolyte is critical to induce crystal change of cathode materials and results in a battery thermal failure.^[^ ^22^ ^]^ In addition, ex situ characterization such as liquid‐nitrogen (LN)‐ceased thermal runaway (TR) test was employed to investigate structural features of battery materials.^[^ ^116^ ^]^ Specifically, liquid nitrogen was added into the ARC chamber to freeze the battery upon the battery voltage dropped to less than 1 V, followed by the analysis of morphology and composition of battery materials using scanning electron microscopy (SEM)‐energy dispersive spectrometer (EDS). The noncalorimetric characterizations contribute to a comprehensive insight into degradation mechanism in batteries during thermal runaway.

### 4.4 Abuse Test

Abuse tests are indispensable for assessing the safety level of a designed Li‐ion battery system before it is being used in a practical application.^[^ ^117^ ^]^ Several types of abuse test have been proposed to investigate the reliability of Li‐ion battery with nonflammable electrolyte: 1) Mechanical abuse (cush, penetration, rollover, etc.^[^ ^117^ ^]^), for example, a nail penetration test of 5 Ah graphite||NMC811 pouch cell using LiPF_6_‐ tris(2,2,2‐trifluoroethyl) phosphate (TFEP)/fluoroethylene carbonate (FEMC) was employed to show the high safety of the designed electrolyte.^[^ ^86b^ ^]^ 2) Electrical abuse (overcharge, over discharge, etc.^[^ ^117^ ^]^), an overcharge test of graphite‐SiO||NMC811 cell with 1 m LiPF_6_ in FEC/DMC (3:7, volume ratio) electrolyte presents a delayed thermal runaway and lower peak temperature than that with commercial 1 m LiPF_6_ in EC/EMC, verifying the higher battery safety with FEC addition.^[^ ^118^ ^]^ 3) Thermal abuse such as overheating, for instance, a heating test was conducted for a graphite||NMC532 battery with a nonflammable concentrated LiFSI/TMP (1:1.9 by molar) electrolyte, the observation of a violent flame indicates that the a low flammability of electrolyte cannot guarantee the thermal safety of full battery.^[^ ^101^ ^]^

## 5 Conclusion and Perspective

**Figure 9:**
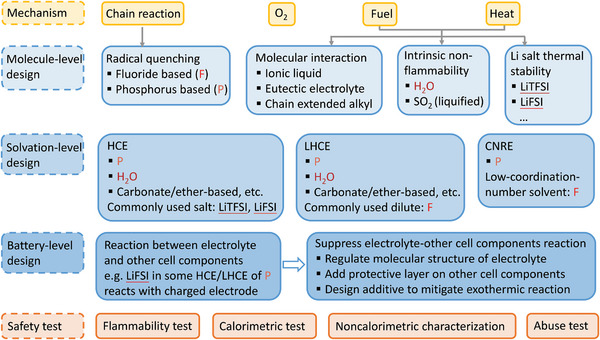
Summary of the discussion of nonflammable liquid electrolyte for safe Li‐ion battery. The molecular design of radical quenching is based on the prevention of chain reaction, the design on molecular interaction, intrinsic nonflammability, and Li salt thermal stability is relevant to the suppression of fuel and heat. Here, fluoride (F)‐, phosphorus (P), H_2_O‐based solvent/additive, and LiFSI, LiTFSI salt are highlighted owing to their research popularity.

We discussed current understanding about thermal runaway mechanism of Li‐ion battery, molecule‐, solvation‐, battery‐level design on nonflammable liquid electrolyte, and safety test for a deeper mechanistic investigation as well as practical application (**Figure** **9**). More effort is required for comprehensive study and effective design on high‐level safety and cell performance. Here, we propose three future research directions to promote the development of nonflammable liquid electrolyte for safe and stable Li‐ion battery.

1. Deeper mechanistic study on thermal runaway is important for a more efficient safety design. Battery fires are intrinsically chain reactions with fire triangle^[^ ^65^ ^]^: i) heat from exothermic reactions or external heating; ii) oxygen from cathode or external air; iii) fuel from flammable electrolyte, reductive gas or charged anode. Combined investigation and design on the suppression of heat, oxygen, fuel, or prevention of chain reaction will improve battery safety efficiently.
2. In addition to battery safety and cycle stability, environmental, and economic considerations are needed for practical applications. For example, although several fluorinated‐based components are effective as flame retardant, high‐thermal stable salt, dilute, etc., lowering fluorinate content or replacing them with nonfluorinated candidates is critical based on cost and toxicity issues.
3. Comprehensive tests on both material‐ and battery‐level are essential for verifying safety, especially a deeper investigation of safety on the battery compatibility of nonflammable electrolytes is indispensable. In addition, safety test and characterization of the battery/material after different cycle numbers with various state of charge, charging/discharging rate are encouraged to provide a more reliable evaluation for practical application.

## Conflict of Interest

The authors declare no conflict of interest.
